# NRAS mutant E132K identified in young-onset sporadic colorectal cancer and the canonical mutants G12D and Q61K affect distinct oncogenic phenotypes

**DOI:** 10.1038/s41598-020-67796-8

**Published:** 2020-07-03

**Authors:** Ryan Timothy D. Yu, Reynaldo L. Garcia

**Affiliations:** Disease Molecular Biology and Epigenetics Laboratory, National Institute of Molecular Biology and Biotechnology, University of the Philippines Diliman, Ma. Regidor St., National Science Complex, 1101 Quezon City, Philippines

**Keywords:** Oncogenes, Oncogenes

## Abstract

Recent data show a global increase in colorectal cancer (CRC) cases among younger demographics, which portends poorer prognosis. The cause of rising incidence is uncertain, and its mutational landscape remains largely unexplored, including those in genes of the epidermal growth factor receptor pathway. Among these are NRAS mutants where there is paucity of functional studies compared to KRAS. Here, the novel NRAS mutant E132K, identified in three tumor samples from Filipino young-onset, sporadic colorectal cancer patients, was investigated for its effects on different cancer hallmarks, alongside the NRAS canonical mutants G12D and Q61K which are yet poorly characterized in the context of CRC. The novel NRAS mutant E132K and the canonical G12D and Q61K mutants show resistance to apoptosis, cytoskeletal reorganization, and loss of adhesion. In contrast to activating KRAS mutations, including the analogous KRAS G12D and Q61K mutations, all three NRAS mutants have no apparent effect on cell proliferation and motility. The results highlight the need to characterize isoform- and mutation-specific oncogenic phenotypes which can have repercussions in disease management and choice of therapeutic intervention. Further analyses of young-onset versus late-onset CRC datasets are necessary to qualify NRAS E132K as a biomarker for the young-onset subtype.

## Introduction

In the Philippines, colorectal cancer (CRC) ranks as the fourth most commonly diagnosed cancer in men, and third in women^1^. Young-onset CRC is a subset of this disease defined by patients who are below 50 years old. Although some cases are observed to be familial, most cases are believed to be sporadic^2^. Globally, young-onset CRC was observed to occur in 2–8% of all CRC cases^3^. In the Philippines, however, young-onset CRC accounts for 17.5% of all CRC cases^4^. Further, it is often discovered at a later stage and has a worse prognosis^3,5^. Incidence of young-onset CRC has been continuously increasing since 2000, which contrasts with the decreasing trend of late-onset CRC^6^.

The epidermal growth factor receptor (EGFR) is commonly overexpressed in cancers, including CRC, leading to an oncogenic phenotype^7^. EGFR can signal via the RAS-RAF-MEK-ERK and PI3K-AKT proto-oncogenic pathways, and can affect cell proliferation, metastasis, survival and growth^7,8^. Apart from EGFR, its downstream effectors are also commonly dysregulated in cancer. Notable among these is the GTPase KRAS, which acts as a molecular switch at the apex of several signaling pathways. Its GTP-bound “on” state can only be deactivated with the binding of a GTPase-activating protein (GAP). Mutations in codons 12 and 13 of this gene, however, inhibit RAS-GAP interactions, leaving KRAS in a constitutively active state, and resulting in the transduction of oncogenic signals^9^. Another isoform of RAS, NRAS, was also found to be mutated in 2–5% of CRCs^10,11^. Whereas previous studies have defined KRAS mutant phenotypes to promote cell proliferation, cell motility, and cytoskeletal disorganization^12–15^, NRAS mutants remain to be largely uncharacterized. Current studies on NRAS mutations in CRC are limited to the canonical mutant G12D and its role in promoting cell survival^16^, thus highlighting the need to characterize other mutants of NRAS.

In this study, the phenotypic effects on various cancer hallmarks of the novel NRAS mutant E132K, and the canonical NRAS mutants G12D and Q61K which remain poorly characterized, are described. The NRAS E132K mutation was identified in three tumor samples from a prospective study of Filipino young-onset, sporadic colorectal cancer patients 45 years old and under (D. L. Sacdalan and R.L. Garcia, unpublished results). The mutation has also been reported in three colorectal cancer cases in the Catalogue of Somatic Mutations in Cancer (COSMIC) database^17^. It was confirmed as somatic but has never been functionally characterized.

## Methods

### Cloning and site-directed mutagenesis of wild type NRAS and mutant variants

Total RNA from HK-2 normal kidney cells (ATCC^®^, Manassas, Virginia, USA, Cat. No. CRL-2190^TM^) was extracted using TRIzol^®^ Reagent (Invitrogen; Thermo Fisher Scientific, Inc., Waltham, MA, USA) according to the manufacturer’s protocol. Total RNA (2 μg) was reverse transcribed using M-MLV Reverse Transcriptase (Promega, Madison, WI, USA) according to the manufacturer’s protocol. The resulting first-strand cDNA was used as template to amplify the full-length wildtype NRAS coding region. To generate the NRAS G12D mutant, a long forward primer incorporating the desired mutation, NRAS G12D-F, was used. To generate the NRAS Q61K and NRAS E132K mutant constructs, splicing-by-overlap-extension PCR was used with requisite primers incorporating the desired mutations. The PCR primers used are listed in Table 1.

**Table 1:** Primers used to generate the wild type and mutant NRAS constructs.

NRAS WT-F	5′-ATGACTGAGTACAAACTGGTGGTGGTTGGAG-3′
NRAS WT-R	5′-TTACATCACCACACATGGCAATCCCATAC-3′
NRAS G12D-F	5′-ATGACTGAGTACAAACTGGTGGTGGTTGGAGCAGATGGT-3′
NRAS Q61K-F (internal)	5′-GACATACTGGATACAGCTGGAAAAGAAGAGTACAGTGC-3′
NRAS Q61K-R (internal)	5′-GCACTGTACTCTTCTTTTCCAGCTGTATCCAGTATGTC-3′
NRAS E132K-F (internal)	5′-GATACAAAACAAGCCCACAAACTGGCCAAGAGTTAC-3′
NRAS E132K-R (internal)	5′-GTAACTCTTGGCCAGTTTGTGGGCTTGTTTTGTATC-3′

PCR was performed using the Titanium^®^ Taq polymerase (Clontech Laboratories, Inc., Mountain View, CA, USA) PCR kit, in a Veriti™ 96-well Thermal Cycler (Applied Biosystems, Foster City, California, USA). Each 10 μl reaction contained 1X of the Titanium^®^ Taq polymerase buffer, 0.25 mM of each dNTP (Vivantis, Subang Jaya, Selang Darul Ehsan, Malaysia), 2 μM of each primer, 1X Titanium^®^ Taq polymerase, and 10 ng of template DNA. The reaction was initially denatured at 95 °C for 5 min and was followed by 25 cycles of denaturation at 95 °C for 30 s, annealing at 65 °C for 30 s, and extension at 72 °C for 40 s. A final extension step at 72 °C for 10 min was incorporated to allow the addition of A-overhangs in preparation for TA-cloning.

Amplified fragments were TA-cloned into the shuttle vector pGem^®^-T Easy (Promega). These were then digested with EcoRI to append the NRAS genes with an EcoRI sequence. These were finally cloned into the mammalian expression vector pTargeT™ (Promega). All clones were checked for directionality and verified error-free by sequencing.

### Cell culture and transfection of NIH3T3/HCT116 cells

Two main cell lines were used for the functional assays—HCT116 human epithelial colorectal carcinoma cells (ATCC^®^; Cat. No. CCL-247TM) and NIH3T3 *Mus musculus* embryonic fibroblast cells (ATCC^®^; Cat. No. CRL-1658TM). The NIH3T3 cell line is often used for characterizing RAS mutants since it does not require cooperative mutations for oncogenic transformation to manifest^18^. For other cancer hallmarks, it was more appropriate to use HCT116, specifically for assays requiring an epithelial phenotype. While this cell line has a KRAS G13D mutant background, transfected oncogenes are still able to modulate its oncogenic readout^19^. HCT116 cells were maintained in Roswell Park Memorial Institute (RPMI) 1640 media (Gibco; Thermo Fisher Scientific, Inc., Waltham, MA, USA) with 10% Fetal Bovine Serum (FBS; Gibco) while NIH3T3 cells were maintained in Dulbecco’s Modified Eagle Medium (DMEM; Gibco) supplemented with 10% New Born Calf Serum (NBCS; Gibco). Both cell lines were incubated in a humidified atmosphere containing 5% CO_2_ at 37 °C and passaged at 80% confluence. Cells were seeded onto either 12-well or 24-well polypropylene tissue culture plates. At 60% confluency, cells in each well were transfected with the appropriate pTargeT™ construct, using LipofectAMINE™ 2000 (Invitrogen). A parallel transfection with the vector pmR-ZsGreen1 (Clontech), which expresses a green fluorescent protein gene reporter, was also done to approximate transfection efficiency. Transfection efficiency of 70–80% was routinely achieved. Cells were harvested at the appropriate times for downstream functional assays.

### Apoptosis assay

Twenty-four hours post-transfection, HCT116 cells were reseeded into a 96-well plate at a density of 10,000 cells per well and were allowed to attach overnight. Once attached, the cells’ media was changed to low serum RPMI 1640 (4% FBS) supplemented with 6 mM sodium butyrate. A parallel setup was run, and this counted as the uninduced setup since its media lacked the apoptotic inducer, sodium butyrate. The plate was wrapped in aluminum foil, and was incubated at 37 °C, 5% CO_2_ for 20 h. After 20 h, 10 µl of the Caspase-Glo^®^ 3/7 Reagent (Promega) was added into each well, and the plate was slightly agitated for 2.5 h in the dark. Once done, the supernatant was transferred to a 96-well flat bottom white plate and was read using a luminescence reader (Varioskan^®^ Flash Spectral Scanning Multimode Reader, Thermo Scientific, Waltham, Massachusetts, USA). To assess apoptosis in each setup, the luminescence readings obtained from the induced setup was normalized against the luminescence readings obtained in the uninduced setup, allowing the values to be comparable across setups.

### Cell proliferation assay

CellTiter 96^®^ AQueous One Solution Cell Proliferation Assay (Promega) was used on HCT116 and NIH3T3 cells to assess the effect of NRAS mutants on proliferative capacity. Cells were initially seeded in 12-well plates, transfected, and reseeded into three 96-well plates, to be used for each day of the assay, with the first day starting at 48 h post-transfection. For each day, fresh standards were made using HCT116 or NIH3T3 cells. These cells were first trypsinized and counted using the TC20™ Cell Counter (Bio-Rad Laboratories, Inc., Hercules, CA, USA), after which predetermined amounts of cells (Day 1: 40,000 cells, Day 2: 80,000 cells, Day 3: 120,000 cells) were seeded into three wells to serve as standards. Each well was then serial diluted two-fold five times to make triplicates of the standards. After the cells were ready, 10 µl of the CellTiter 96^®^ AQueous One reagent was added into each well, and the plate was incubated at 37 °C, 5% CO_2_ for 30 min to an hour. The absorbance of each well at 492 nm was then read using a colorimetric plate reader (FLUOstar Omega Microplate Reader, BMG Labtech, Cary, NC, USA). A standard curve was generated using the linear relationship of the standards’ absorbance and their cell count, and the equation of the line was used to calculate the individual cell counts of the samples. The same was done for days 2 and 3 to be able to plot the cell count of each sample against time.

### Scratch wound assay

Upon reaching 80% confluence for HCT116 cells, or full confluence for NIH3T3 cells, a sterile yellow micropipette tip was used to scratch the cell monolayer in a straight line. The media was then changed to low serum media (RPMI 4% FBS or DMEM 2.5% NBCS, respectively). The cells were incubated at 37 °C, 5% CO_2_, and pictures of the same spot were taken immediately after scratching and 18 h post-scratch. To calculate the percentage of open area that remained, three random points per scratch were selected. The distance between the two migrating fronts at these three points were then measured using Adobe Photoshop CC 2014 (Adobe, Inc.; San Jose, California, USA) at the starting and finishing time. The values obtained at the finishing time were then divided by the values obtained at the starting time to get the percentage of open area remaining.

### Visualization of changes in cytoskeletal organization

Effects of NRAS mutants on cytoskeletal structure were observed through actin staining. NIH3T3 cells were transfected in a 24-well plate and reseeded into a Millicell EZ Slide 8-well glass chamber slide (Millipore Sigma; Burlington, Massachusetts, United States) 24 h after. Cells were allowed to attach for 24–36 h prior to being fixed with ice-cold 4% paraformaldehyde for 20 min with shaking at 40 rpm on ice. The cells were then washed three times with 200 µl of 1 × PBS before being permeabilized with 200 µl of cold 0.1% Triton X-100 in 1 × PBS, with shaking at 40 rpm for 5 min in room temperature. The cells were then blocked with 1% (w/v) bovine serum albumin (BSA) in 1 × PBS for 20 min while shaking at 40 rpm in room temperature. The blocking solution was removed after which the cells were washed three times with 1 × PBS. All subsequent steps were performed in the dark. For actin staining, 100 µl of 0.165 µM Alexa Fluor^®^ Phalloidin (Life Technologies, Thermo Fisher Scientific, Inc., Waltham, MA, USA) was added into each well for 30 min at 40 rpm in room temperature. The cells were washed three more times before being counterstained with 100 µl of 5 µg/ml Hoechst^®^ 33342 (Life Technologies) for 5 min in room temperature. Another PBS wash was performed before removing the chamber slide barrier and covering the slide with a 0.13 mm glass coverslip. Slides were viewed under an IX83 Inverted Microscope (Olympus Corp., Tokyo, Japan) using the green fluorescent filter (λex/λem: 490/525 nm) to visualize stained filamentous actin, and the blue fluorescent filter (λex/λem: 355/465 nm) to observe the nucleus at 400X magnification.

### Monitoring levels of E-cadherin by western blot analysis

Twenty-four hours post-transfection, the media for HCT116 cells was replaced with low serum RPMI 1640 (4% FBS). Total protein was extracted using radioimmunoprecipitation assay (RIPA) lysis buffer [150 mM NaCl, 0.5% sodium deoxycholate, 0.1% sodium dodecyl sulphate (SDS), 50 mM Tris (pH 8.0) supplemented with protease inhibitors (1 mM phenylmethylsulfonyl fluoride (PMSF), 5 mM EDTA, and 10 μM E64 (Roche Diagnostics GmbH, Mannheim, Germany)]. Lysates were cleared by centrifugation at 10,000 g for 20 min at 4 °C. Total protein concentration was quantified using the bicinchoninic acid (BCA) assay (Sigma-Aldrich, St. Louis, Missouri, USA) according to the manufacturer’s protocol. Lysates were stored at -80˚C until use. Each sample was then loaded into a Mini-PROTEAN^®^ TGX Stain-Free™ (Bio-Rad) polyacrylamide gel and run at a constant 200 V for 20–30 min. Gels were blotted onto a Trans-Blot^®^ Turbo™ Mini PVDF membrane using the Trans-Blot^®^ Turbo™ Blotting System (Bio-Rad) using a pre-set protocol: Mixed MW. Membranes were incubated overnight in primary antibody solution (1:5,000 dilution of antibody in blocking solution) at 4 °C. The following antibodies were used: E-cadherin (mouse α-E-cadherin polyclonal antibody; EMD Millipore, Burlington, MA, USA; Cat no. 07-697), GAPDH (mouse α-GAPDH, Calbiochem, San Diego, California, USA; Cat no. CB1001), and NRAS (rabbit α-NRAS polyclonal antibody; Invitrogen; Cat no. PA5-34560). After incubation with a freshly prepared secondary antibody solution [1:10,000 dilution of the antibody, 1:10,000 Precision Protein™ StrepTactin-HRP conjugate (Bio-Rad) in blocking solution] for 1 h, bands were visualized with enhanced chemiluminescence (ECL) substrate (Merck, Kenilworth, New Jersey, USA) and imaged using the ChemiDoc Touch Imaging System (Bio-Rad). The following secondary antibodies were used: HRP-conjugated goat α-mouse IgG (ThermoFisher Scientific Inc.; Cat no. 31430) and HRP-conjugated goat α-rabbit IgG (ThermoFisher Scientific Inc.; Cat no. 31460).

### Immunocytochemistry

To assess the expression and localization of E-cadherin in individual cells, immunocytochemistry for E-cadherin was performed on transfected HCT116 cells. Twenty-four hours post-transfection, 10,000 cells were re-seeded into 96-well plates, and grown to 80% confluence over 48 h. Cells were then fixed with ice-cold 100% methanol at − 20 °C for 15 min. Cells were washed three times with cold 1 × PBS then permeabilized with 100 µl of 0.1% Triton X-100 in 1 × PBS for 5 min while shaking at 60 rpm in room temperature. Cells were again washed three times in 1 × PBS followed by a blocking step with 100 µl of 1% BSA in 1 × PBS for 30 min at 60 rpm in room temperature. The blocking solution was removed and replaced with 100 µl of the E-cadherin 1° antibody (1:1,000 in 1% BSA; ThermoFisher Scientific Inc.; Cat no. MA5-11496), then incubated for 2 h at 60 rpm in room temperature. This was followed by three more washes with cold 1 × PBS, prior to adding the Cy5-conjugated goat α-mouse secondary antibody (1:400 in 1% BSA; Merck; Cat no. AC111S) in the dark. The cells were washed three more times with cold 1 × PBS before being counterstained with 50 µl of 5 µg/ml Hoechst^®^ 33342 (Life Technologies) for 5 min while shaking at 60 rpm. Cells were once again washed with 1X PBS three times, before adding the mounting solution, 100 µl of 70% glycerol. The cells were visualized using the IN Cell Analyzer 6000 (GE Healthcare Life Sciences; Marlborough, Massachusetts, USA) at 400 × magnification.

### Protein modelling

To assess the impact of the single amino acid mutations on protein structure, NRAS Q61K and E132K were first modelled using the SWISS-MODEL web-based client^20,21^. BIOVIA Discovery Studios Visualizer was then used to compare the modelled NRAS mutants with the crystal structure of wild-type NRAS (PDB: 5UHV)^22^. The NRAS mutant structures were first superimposed with reference to the NRAS WT crystal structure. The overlay similarity value was obtained from the resulting data table. The two proteins were then superimposed via their C-alpha pairs. From this, a report was generated showing the global root-mean-square deviation.

### Statistical analyses

All quantitative data were presented as mean ± standard error (SE). The Student t-test was used to compare the NRAS mutant setups against NRAS WT or the empty vector setup.

### Ethical approval

This article does not contain any studies with human participants or animals performed by any of the authors. The mutant gene constructs described were generated from cloned wild type templates available in the laboratory.

## Results

### Effects of NRAS mutants G12D, Q61K and E132K on cell survival

**Figure 1:**
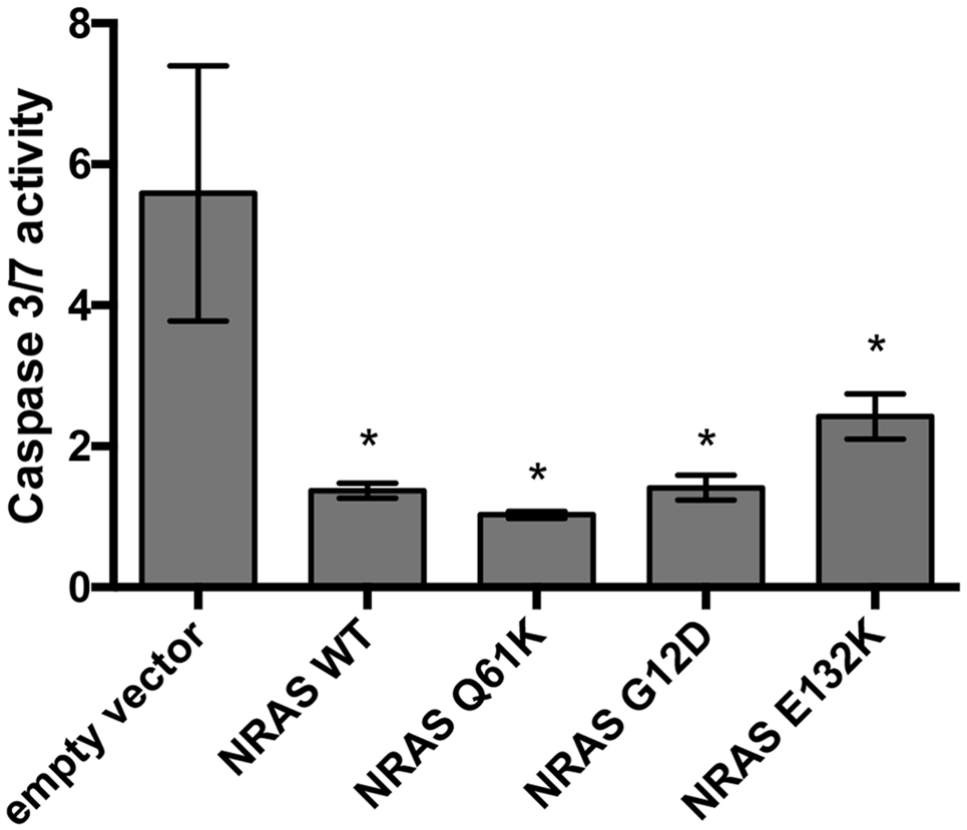
Overexpression of NRAS WT and mutants promotes cell survival in HCT116 cells. Transfected HCT116 cells were assayed for their ability to resist apoptosis upon induction with 6 mM sodium butyrate. Cells that overexpress NRAS WT and NRAS mutants significantly promoted cell survival in comparison to the empty vector set-up (*p < 0.05). Representative of three trials in triplicates.

Activation of the MAPK and PI3K/AKT pathways typically confers resistance to apoptosis. The anti-apoptotic effects of NRAS mutants were assessed through the Caspase-Glo^®^ 3/7 assay. The assay measures the amount of caspase 3 and caspase 7 present in the cell via a DEVD-aminoluciferin substrate. This substrate can only be consumed by luciferase once caspase 3 or caspase 7 cleaves the DEVD peptide off, which allows for the measurement of caspase 3 and 7 activity with a luminescence readout. Transfected cells were initially induced to apoptose using 6 mmol of sodium butyrate, which has been previously documented to induce both extrinsic and intrinsic pathways of apoptosis, to observe which cells can resist the apoptotic stimuli more^23^. Based on the results obtained, NRAS WT, G12D, Q61K, and E132K, when overexpressed, all promote cell survival compared to vector-only control (Fig. 1). Despite the wild-type gene not being constitutively active, it seemed that the mitogenic stimulus in low serum media (4% FBS) was enough to activate wild-type NRAS, such that it reaches the threshold needed to promote cell survival. These results are consistent with those reported in the literature^12,16,24,25^, which showed that NRAS G12D can confer resistance to apoptosis while having no effect on proliferation in CRC in vivo.

### NRAS G12D, Q61K and E132K have no impact on cell proliferation in HCT116 and NIH3T3 cells

**Figure 2:**
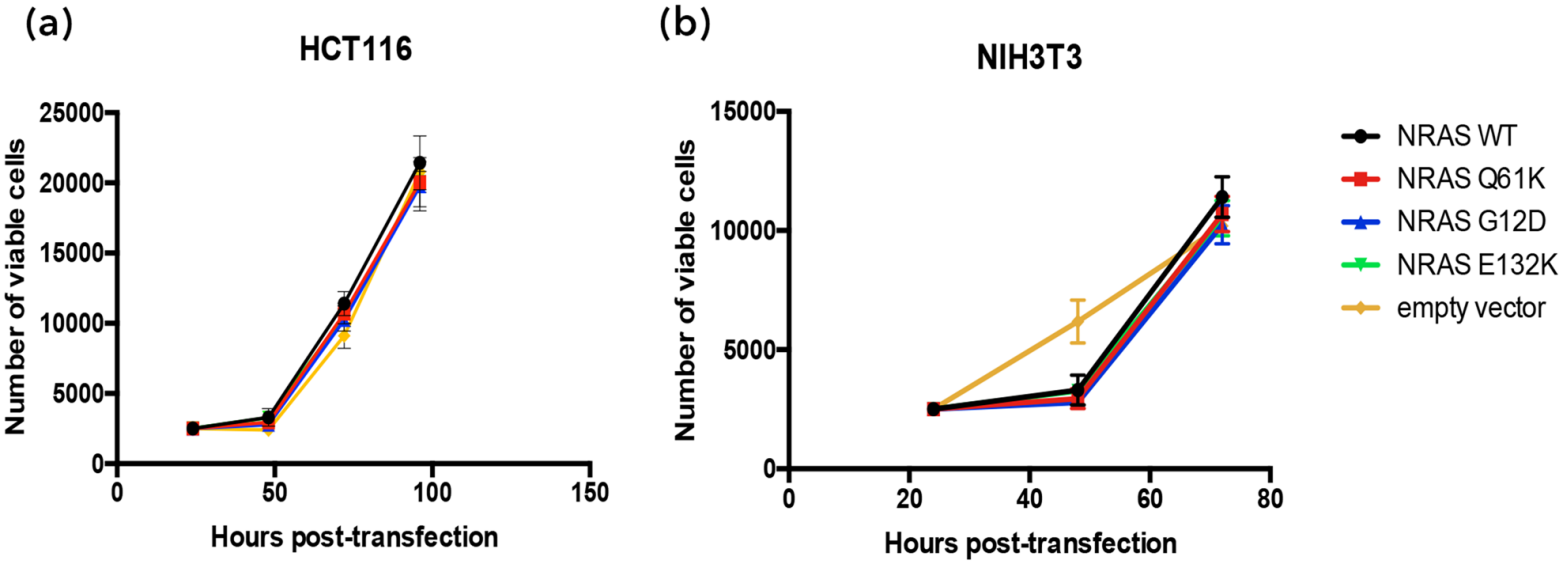
NRAS G12D, Q61K and E132K do not affect proliferative capacity of HCT116 and NIH3T3 cells. HCT116 cells were transfected with NRAS wild type or mutant constructs and assayed for proliferative capacity using the MTS assay. An equal number of transfected cells were seeded into three 96-well plates and were grown in low serum conditions (4% FBS) for three days. Cell standards were seeded everyday to measure the samples’ cell count, and the cell count for each day was then plotted for the different samples.

The effects of NRAS G12D, Q61K and E132K on the proliferative capacity of HCT116 and NIH3T3 cells were assessed using the CellTiter96 Aqueous One Cell Assay, an assay that indirectly counts cells based on their ability to metabolize the MTS tetrazolium into a colored formazan product, the absorbance of which is measured. After taking the cell counts for three separate days, the transfected HCT116 and NIH3T3 cells’ proliferation was plotted (Fig. 2) and no significant differences in proliferation were observed between the NRAS mutants and the empty vector control. NRAS G12D was also found to have no effect on proliferation in colorectal cancer in vivo^12,16^.

### Cell motility

**Figure 3:**
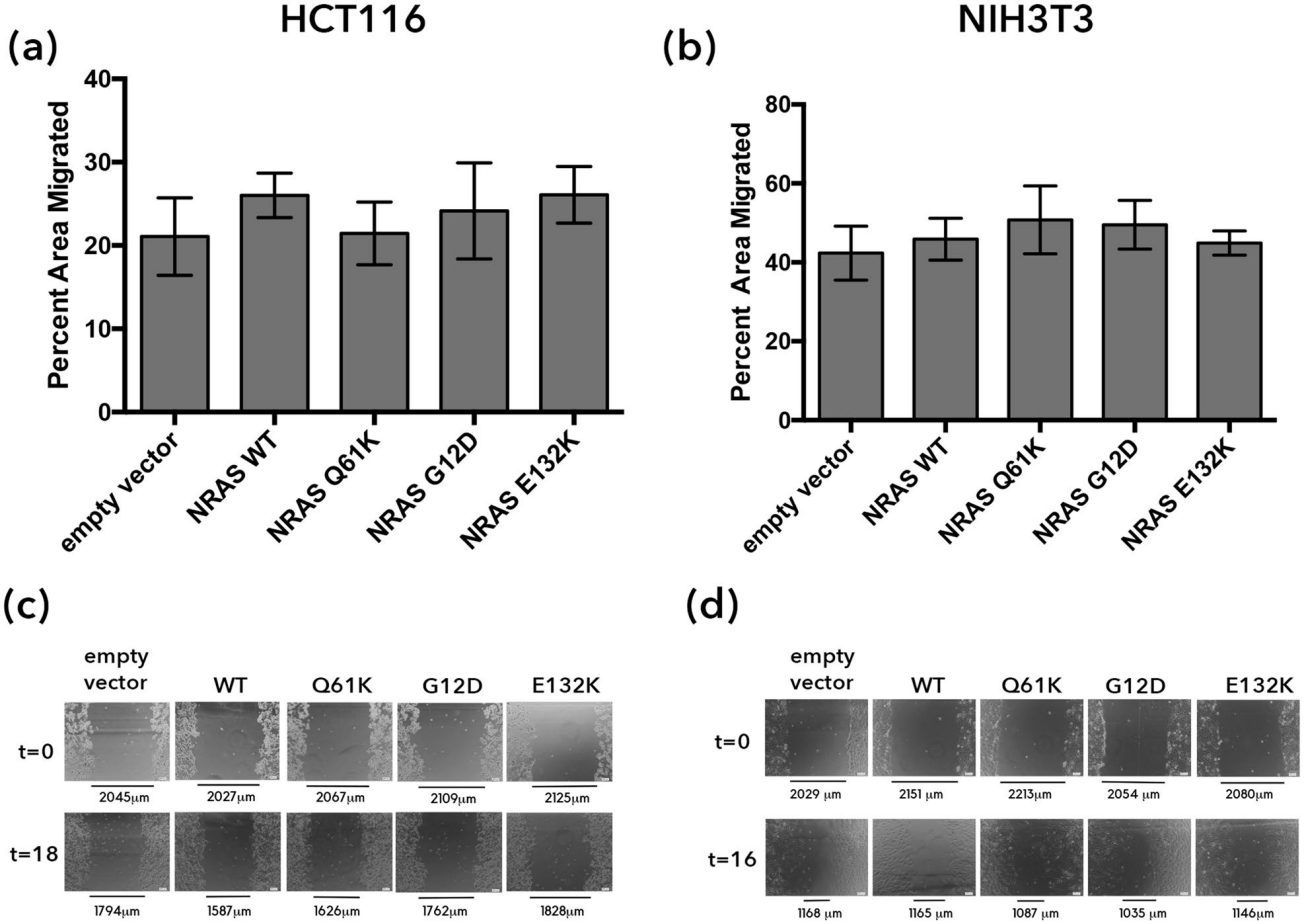
Overexpression of NRAS mutants does not affect cell motility in HCT116 and NIH3T3 cells. The width of the open area of each set-up was measured at two time points, 0 h and 18 h for HCT116 and 0 and 16 h for NIH3T3 cells, and the percent open area of each sample were plotted against each other. No significant changes in terms of motility were observed in HCT116 (**a**, **b**) and NIH3T3 (**c**, **d**) cells.

To assess the effects of NRAS G12D, Q61K and E132K on cellular motility, wound healing assays were performed in both HCT116 and NIH3T3 cells. This technique is used to measure the migration rate of cells by observing the movement of cells into the created wound. No significant differences in migration rate between the NRAS mutants and the empty vector setup were observed in transfected HCT116 (Fig. 3a) under low serum conditions (4% FBS). However, this may be because HCT116 is a cancer cell line, and NRAS overexpression can no longer augment its migratory capacity. To verify if indeed NRAS mutants have no effect on cellular migration, the assays were also performed in NIH3T3 cells (Fig. 3b) under low serum conditions (2.5% NBCS). Similar results were obtained suggesting that wild type and the canonical NRAS mutants G12D and Q61K, as well as the novel mutant E132K, have no effect on cellular migration.

### NRAS G12D, Q61K, and E132K mutants promote morphological polarization and cytoskeletal reorganization in NIH3T3 cells

**Figure 4:**
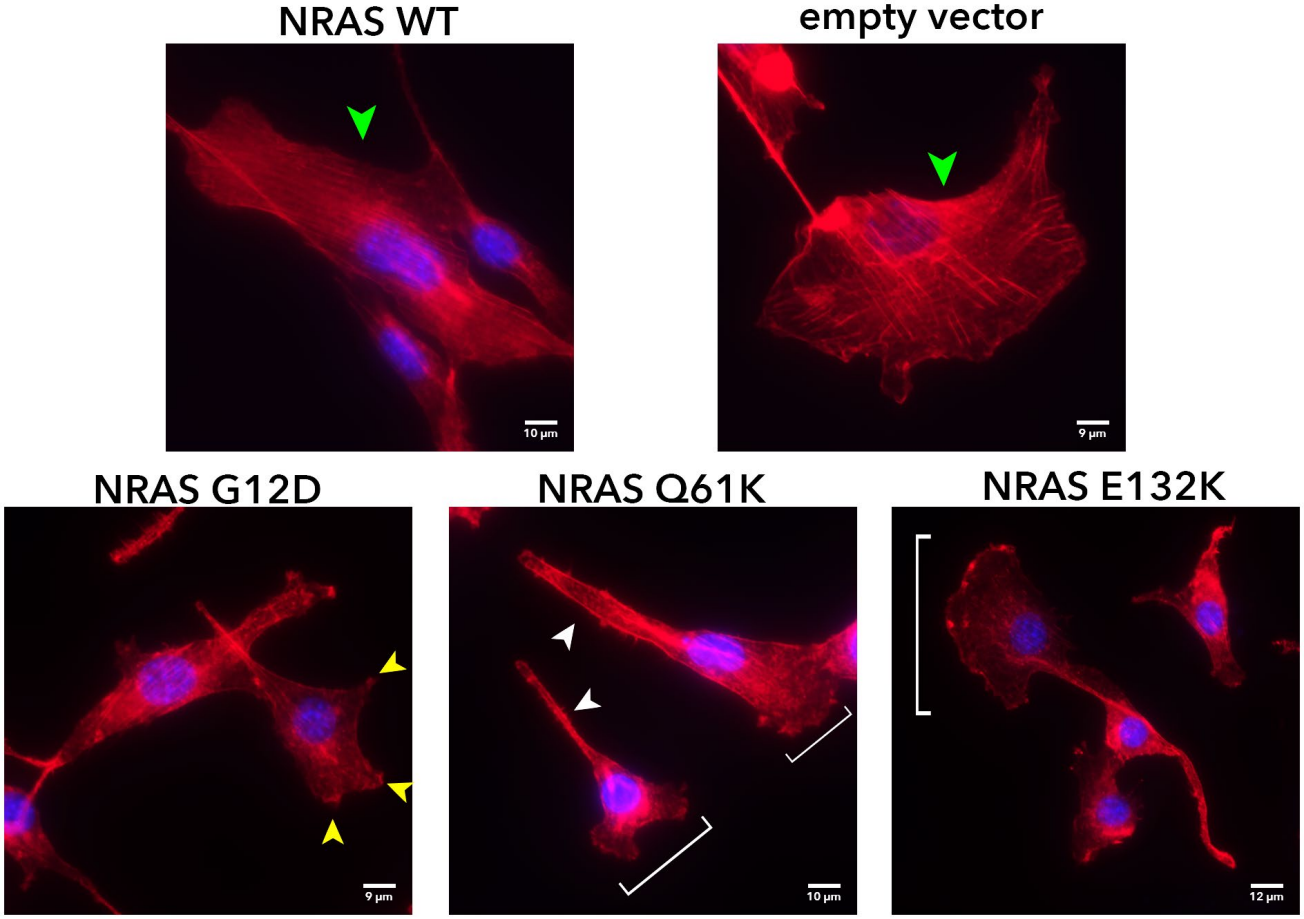
NRAS G12D, Q61K and E132K promote cytoskeletal reorganization in NIH3T3 cells. Cytoskeletal actin organization of transfected NIH3T3 cells was observed after staining with fluorescence conjugated phalloidin. Green arrowheads point to actin filaments. Yellow arrowheads point to lamellipodia. White arrowheads point to filopodia. White brackets point to highly polarized cells with convex leading edge, suggestive of a migratory phenotype.

Changes in cytoskeletal organization were monitored by staining using Alexa Fluor™ 594-conjugated or Alexa Fluor™ 488-conjugated phalloidin, which strongly binds to F-actin within the cell, staining it red or green, respectively. For this purpose, NIH3T3 cells were used as they can more vividly show detailed changes in cytoskeletal organization. Drastic changes in the cytoskeleton were observed when NRAS G12D, Q61K, and E132K were overexpressed. Whereas cells transfected with NRAS WT or empty vector control have a larger cytoplasm and a parallel array of stress fibers, cells transfected with the NRAS mutants exhibited a shrunken cytoplasm, and an increase in filopodia and long protrusions (Fig. 4). Structures such as sheet-like lamellipodia, finger-like pseudopodia and needle-like filopodia point to a migratory phenotype^26–28^. Further, cells transfected with NRAS mutants showed more polarized cells, evident in the uneven distribution of F-actin in the cell, and the conical shape with convex leading edge in the case of NRAS Q61K and E132K. All these suggest that the NRAS mutants G12D, Q61K, and E132K promote migratory capacity.

### NRAS G12D, Q61K, and E132K negatively regulate E-cadherin expression

**Figure 5:**
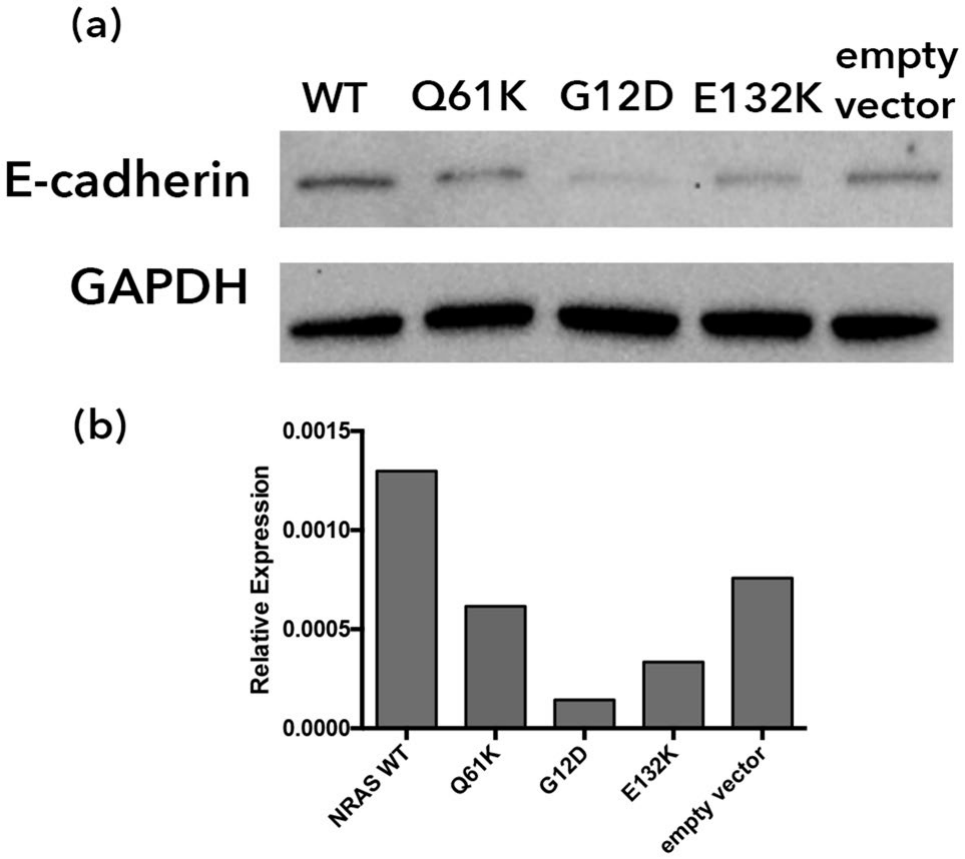
Western blot analysis of transfected HCT116 cells shows NRAS mutants downregulate E-cadherin expression. (**a**) Western blot for E-cadherin on transfected HCT116 cells was performed 72 h post-transfection with GAPDH as a loading control. (**b**) A densitometric analysis was performed to visualize changes in E-cadherin expression. Full-length blots are presented in Supplementary Fig. 1.

The effects of NRAS G12D, Q61K, and E132K on the expression of E-cadherin in HCT116 cells were examined through western blot analysis and immunocytochemistry. E-cadherin is primarily found in adherens junctions^29,30^. It is often downregulated or mutated in carcinomas, and diminished expression is correlated with loss of epithelial features and a more metastatic phenotype^31^. Western blot analysis (Fig. 5, Supplementary Fig. 1) showed downregulation of E-cadherin in cells transfected with NRAS G12D, Q61K and E132K, compared to wild type which is indicative of loss of cell adhesion.

**Figure 6:**
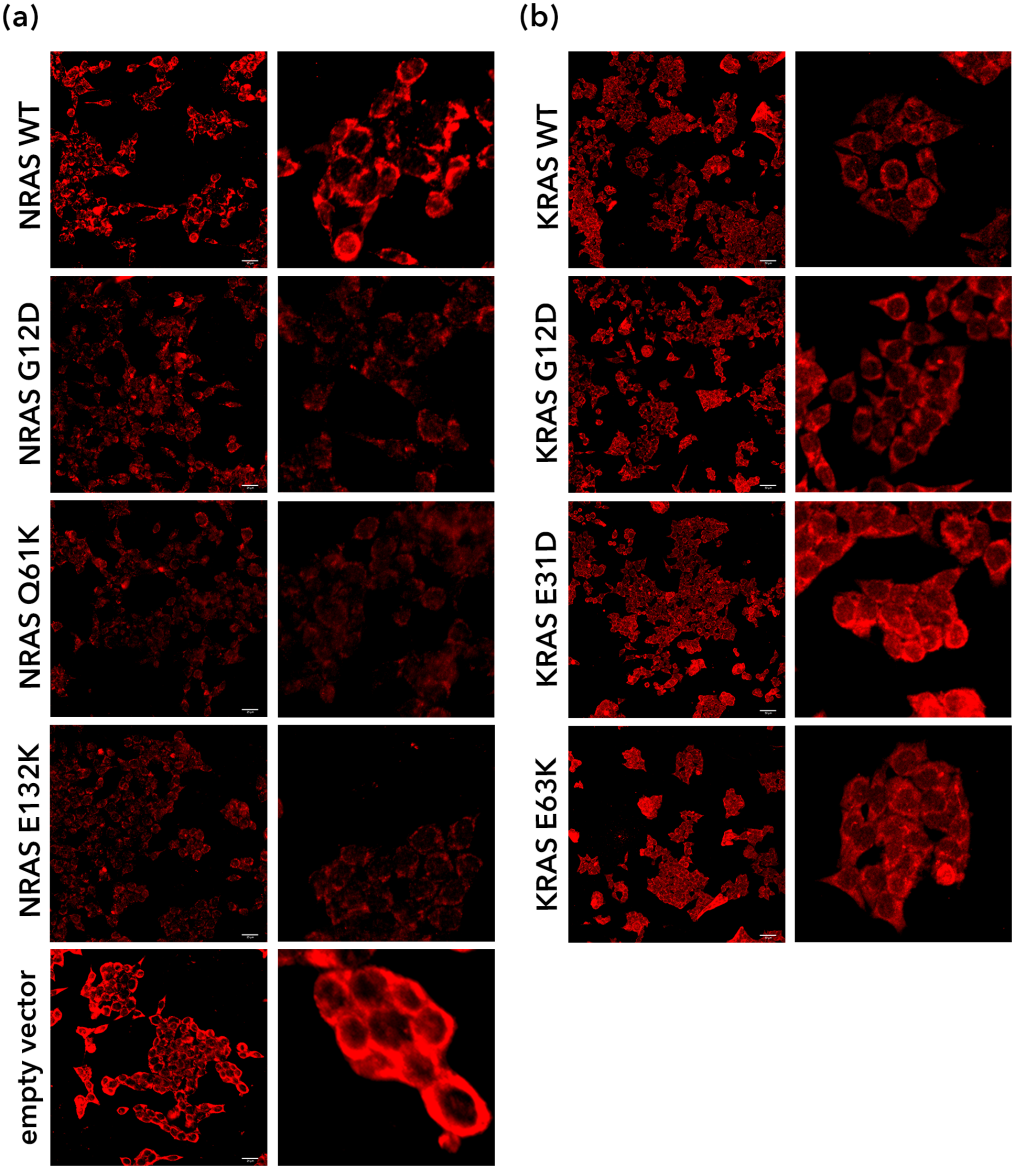
NRAS G12D-, NRAS Q61K- and NRAS E132K-transfected HCT116 cells have altered E-cadherin expression and localization. Immunocytochemical staining for E-cadherin was performed on HCT116 cells transfected with (**a**) NRAS and (**b**) KRAS constructs. Images were zoomed in (right hand panels) to better visualize the punctate or solid E-cadherin rings.

To confirm that there is indeed a loss of cell adhesion in cells transfected with NRAS mutants, immunocytochemical staining of E-cadherin was carried out. HCT116 cells transfected with NRAS WT, G12D, Q61K and E132K all showed a hazy or punctate ring of E-cadherin surrounding the cell, in contrast to cells transfected with the empty vector control, which appear to have a prominent ring of E-cadherin (Fig. 6). Additionally, despite the high confluence of cells, they did not appear to adhere to each other or form clumps. While there were regions of intense staining in NRAS WT-transfected cells, they did not have the solid ring of E-cadherin observed in the vector-only control. HCT116 cells transfected with wild type KRAS, canonical KRAS mutant G12D, and the novel KRAS mutants E31D and E63K^15^, on the other hand, all showed more intact E-cadherin rings similar to the empty vector control.

### In silico protein modeling of the novel mutant NRAS E132K

**Figure 7:**
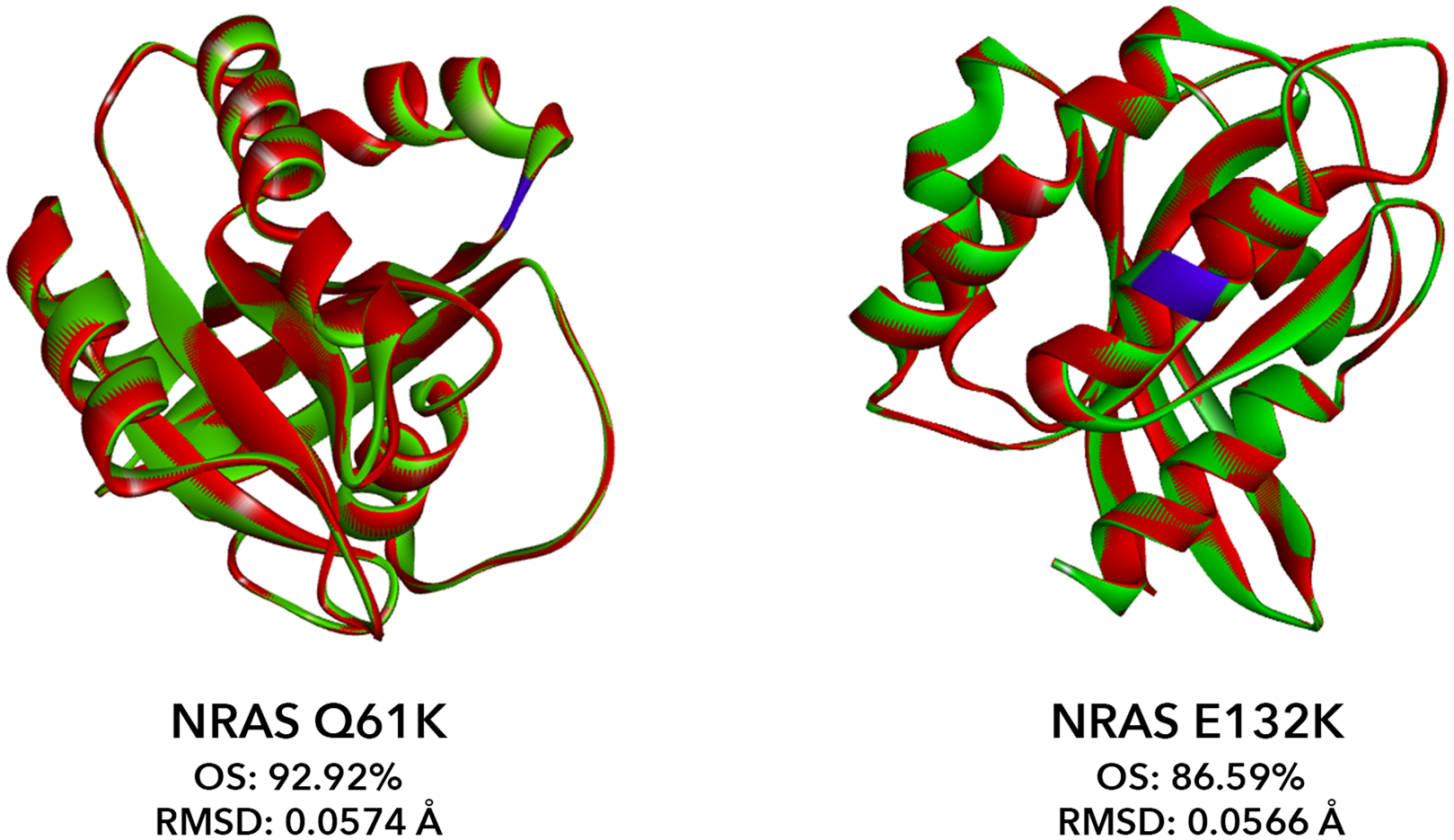
In silico structural analysis of NRAS Q61K and E132K with reference to NRAS WT. Protein models of the NRAS mutants Q61K and E132K were generated using SWISS-MODEL (red) and were superimposed to the crystal structure of NRAS WT (green). The corresponding point mutation for each mutant is centered and highlighted blue. The overlay similarity (OS) score and root-mean-square-deviation (RMSD) were calculated using BIOVIA Discovery Studios Visualizer.

In silico protein modeling analysis was performed on NRAS Q61K and E132K by comparing their protein structures with that of NRAS WT (PDB: 5UHV). An overlay similarity (OS) score of 92.92% and root-mean-square deviation (RMSD) of 0.0574 Å was obtained for NRAS Q61K while an OS score of 86.59% and an RMSD of 0.0566 Å was obtained for NRAS E132K (Fig. 7). The values obtained suggest that NRAS Q61K and E132K do not have an overall significant effect on the structure of NRAS. This may be an important feature of RAS activating mutations, as the function of the RAS protein has to be conserved in order to be hyperactive.

Despite not having a significant global impact on NRAS protein structure, NRAS G12D and Q61K have been shown to cause oncogenicity by inhibiting GTP hydrolysis. The glycine residue at codon 12 sits at a pocket where GAP interacts with RAS^32^. By substituting glycine with an amino acid with a bulkier side chain, this interaction is inhibited, and the GTP hydrolysis is hindered^33–35^. Similarly, Q61 is located in a crucial position that activates the water molecule necessary for GTP hydrolysis. As glutamate is the only amino acid that can function in activating the water molecule, any mutation to codon 61 may disrupt GTP hydrolysis. NRAS E132K was only recently identified in a large-scale sequencing study involving 13,336 CRC tumors^36^ but its functional consequences in vitro and in vivo have never been described. The affected amino acid is located in the α-helix bordered by two amino acid motifs (G4, NKXD and G5, EXSAK) that are deemed important for binding the guanine base of the nucleotide^22,36^. Despite not having significant effects on protein structure, the acidic to basic amino acid change in NRAS E132K was found to affect distinct cancer hallmarks.

## Discussion

Since the late 1970s, CRC patients have benefitted a great deal from screening efforts, which were thought to have led to the decrease in CRC incidence^6^. However, regular screening is usually advised to begin at the age of 50; thus, leaving out 2–8% of the population that acquire young-onset CRC^3,6^. Currently, the cause for the rising incidence of young-onset CRC is uncertain, but there is evidence to show that the rise is not solely a result of more detection^37^.

Some cases of young-onset CRC are usually associated with hereditary forms, including Lynch syndrome, classic familial adenomatous polyposis, and juvenile polyposis syndrome^6,38,39^. The NRAS mutants characterized here were identified from a prospective study of young-onset CRC cases (D. L. Sacdalan and R.L. Garcia, unpublished). Familial CRC was ruled out by screening only tumor samples with functional MLH1, MSH2, MSH6, or PMS2. The expression of these mismatch repair genes is characteristically downregulated in Lynch syndrome, which is the most common form of hereditary CRC^6^.

Young-onset CRC is observed to be phenotypically different from late-onset CRC. Several reports have documented tumors from patients below 50 years old to often occur in the right colon and the rectum^3,5,6^. As well, less differentiated, mucinous, and signet-ring cell features are often associated with young-onset CRC^5,6^. A more advanced stage during diagnosis is also commonly observed^3,5,6^. All these clinicopathological features have contributed to this disease having a worse prognosis when compared to late-onset CRC^6^. Despite having well-defined features, molecular cellular studies on sporadic young-onset CRC are only in its infancy ^3^. Epigenetic studies have linked young-onset CRC to a CpG island methylator phenotype (CIMP)-low, microsatellite stable (MSS), and LINE-1 hypomethylated phenotype^3^. A study conducted by Hong et al*.*^40^ has also revealed that upregulation of *CYR61, FOS, FOS B, KRT24, VIP, EGR1,* and *UCHL1* is correlated with young-onset CRC. Studies regarding the mutational status of cancer-related genes, however, are lacking.

The EGFR pathway is known to be overexpressed in 60–80% of colorectal cancer, which drives its oncogenicity^7,41–43^. For metastatic colorectal cancer, monoclonal antibody treatments have been used to block EGFR and cause its eventual ingestion. However, many patients harboring KRAS mutations remain refractory to anti-EGFR therapy. Thus, regulatory authorities such as the Food and Drug Administration (FDA) in the United States and the European Medicines Agency (EMA) in Europe, warrant that only those who have been prescreened to be wild type for KRAS should be administered the drug. Still, drug resistance persists in a subset of patients with no known KRAS mutations, prompting the American Society of Clinical Oncology to update its guidelines and recommend the inclusion of non-hotspot mutations in both KRAS and NRAS in diagnostic tests. A number of studies also indicate that mutations in other downstream effectors and regulators of the EGFR pathway, including BRAF, PIK3CA, and PTEN, will have to be functionally characterized to identify additional biomarkers for non-responsiveness to anti-EGFR therapy^44^. Thus far, high-throughput resequencing efforts worldwide aimed at identifying unknown somatic mutations in human cancer have already revealed that yet uncharacterized mutations in KRAS, NRAS, BRAF, PIK3CA, PTEN and other cancer genes are highly prevalent^45–47^. The usefulness of identifying these uncharted mutations, as well as their significance in primary (de novo) and secondary (acquired) modes of resistance to targeted therapies are increasingly being recognized^48,49^. In multiple studies, patients with NRAS mutation were shown to be unresponsive to anti-EGFR treatment, hence the EMA does not recommend the use of anti-EGFR drugs for these patients as well^50^.

The clinical utility of these potential biomarkers in prognostication varies and argues for individual characterization of their effects on patient outcomes^51^. In a study of 3,278 patients with stage II and III colon cancer patients receiving irinotecan added to fluorouracil (FU)/leucovorin (FA) as adjuvant treatment, KRAS mutation did not have significant prognostic value while BRAF was prognostic for overall survival in MSI-low and stable tumors^52^. In a similar study involving 229 patients, BRAF was shown to be prognostic of poor survival in advanced and recurrent CRC^53^. The case of PIK3CA is different depending on the molecular lesion involved. Mutations in the gene have been associated with a significant reduction in survival of CRC patients who are wildtype for BRAF^54^. In contrast, PIK3CA amplification appears to be an independent prognostic marker for patients who may benefit the most from adjuvant therapy^55^. Loss of PTEN is associated with poor prognosis in stage II patients or those with liver metastasis^56,57^. The relevance of characterizing exon-specific NRAS mutations was highlighted in the study of Schirripa et al.^50^. Patients with NRAS mutation had a much lower overall survival (OS) rate compared to wildtype patients (25.6 months vs. 42.7 months) and no differences in survival were observed in patients harboring NRAS and KRAS mutations. More importantly, OS seemed to depend on the position of the mutation in the exons, being shorter in those harboring mutations in exon 3 compared to those who are wild type or have mutations in exon 2 of the gene. Mutations in genes which are part of the same signaling pathway are believed to be mutually exclusive. In the context of tumor heterogeneity, however, it is not totally unlikely to have coexistent mutations coming from different pockets of cells within a tumor.

The NRAS mutants G12D (NM_002524.4, c.35 G > A, p. G12D) and Q61K (NM_002524.4, c.181 C > A, p. Q61K) were included in the study since KRAS has analogous mutations in these positions that are well-characterized to be highly tumorigenic, but those for NRAS are poorly characterized in the context of CRC^12,15,58^. Despite the dearth of functional data, these two mutants are predicted to be pathogenic by the algorithm Functional Analysis Through Hidden Markov Models (FATHMM) with scores of 0.91 and 0.99, respectively. The third mutant, E132K (NM_002524.4, c.394 G > A, p. E132K), was similarly predicted to be pathogenic with a FATHMM score of 0.99. The FATHMM algorithm is a sequence-based tool that scores the functional consequences of mutations^59^. A score greater than 0.70 means it qualifies for ‘pathogenic’ mutations.

The proliferative capacity of NRAS was observed through the MTS Assay over three days. NRAS G12D, Q61K, and E132K were all observed to have no significant effects on proliferation when compared to the empty vector setup. This is in contrast to the known effects of activating KRAS mutants on promoting proliferation^12,13,15^. Despite having analogous mutations in hotspot codons (i.e. G12D and Q61K), it seems that these two isotypes of RAS differ in their ability to promote cellular proliferation.

The effect of activating RAS mutations on cell survival appears to be context-dependent, and is influenced by which RAS isoform is involved, the specific mutation, and the cell type it is expressed in. For instance, HRAS G12V expression in melanoma inhibits apoptosis^60^. In this study, the canonical mutants NRAS G12D and Q61K, as well as the novel mutant E132K , were shown to confer resistance to apoptosis, in stark contrast to the analogous mutations in KRAS (KRAS G12D and KRAS Q61K) which have been shown to promote butyrate-induced apoptosis in HCT116 cells^61^. The repercussions of these distinct oncogenic properties are vast, should KRAS and NRAS mutations coexist within a heterogenous tumor. In a representative scenario, if an anti-proliferative drug is given, cells with an NRAS mutation may escape chemotherapy and survive throughout the course of treatment, becoming the dominant population of cells during relapse.

The metastatic potential of cells transfected with the NRAS constructs was studied through their migratory capacities, cytoskeletal organization, and effects on cell adhesion. The canonical mutants NRAS G12D and Q61K, as well as the novel mutant E132K, failed to show signs of increased cellular motility. This is in contrast to the analogous KRAS mutants (KRAS G12D and KRAS Q61K) as well as other non-hotspot KRAS mutants which have been documented to promote cell migration^14,15^. This further confirms that the two isoforms of RAS may signal through distinct pathways, thereby affecting distinct oncogenic phenotypes. Results obtained from cytochemical staining of actin filaments, however, suggest an increase in migratory capabilities. Cytoskeletal disorganization, morphological polarization and pseudopodial formations are highly suggestive of a migratory phenotype, as these are among the first few steps taken by the cell for it to move^62^. The following steps would include the traction of myosin II with the actin filaments to pull the cell towards the pseudopodia, and also the detachment of the trailing end of the cell to allow the whole cell to move towards the pseudopodia^62^. These results, therefore, suggest that the expression of NRAS G12D, Q61K, and E132K affect the early phases of migration, but not necessarily the latter stages that involve motility.

Aside from morphological polarization and the formation of migratory actin structures, another early event in cell migration is the loss of cell adhesion, which is largely controlled by E-cadherin expression^29,63^. E-cadherin expression was assessed through western blot analysis and immunocytochemistry. In both experiments, HCT116 cells transfected with NRAS G12D, Q61K, and E132K all showed a decrease in E-cadherin expression, suggesting that these mutants can cause loss of cell adhesion. In contrast, when HCT116 cells transfected with KRAS WT, G12D, E31D, and E63K were analyzed via immunocytochemistry, no significant changes were observed when compared to empty vector control.

Based on the results of this study on NRAS mutants and on what is already known about the effects of KRAS mutations on cell motility, cytoskeletal organization, and E-cadherin expression, it is evident that these two isoforms of RAS affect metastasis differently. NRAS G12D, Q61K, and the novel NRAS E132K mutant were all observed to downregulate E-cadherin expression and promote cytoskeletal disorganization, which are consistent with affecting early events during cell migration, while having no effect on motility. In contrast, analogous KRAS mutations in codons 12 and 61, as well as non-canonical and non-hotspot KRAS mutations affect both cellular motility and cytoskeletal disorganization^14,15^. Because these two isoforms of RAS potentially affect different phases in metastasis, coexistence of NRAS and KRAS mutations in cells may lead to a more metastatic phenotype.

In this study, the oncogenic phenotypes of the NRAS mutant E132K, identified from a cohort of Filipino young-onset sporadic CRC patients, were described, and were observed to be similar to those of the poorly characterized canonical mutants NRAS G12D and NRAS Q61K. Despite having analogous mutations in codons 12 and 61, NRAS was observed to play a distinct role in oncogenesis when compared to KRAS. These phenotypic differences are highly relevant considering the emerging mechanisms of chemoresistance and therapeutic failure. These in vitro phenotypic readouts for NRAS G12D, Q61K, and E132K add to and are consistent with the limited in vivo data on NRAS function. While RAS isoform-specific oncogenic effects can be gleaned from this study, investigations on effects of exon-specific mutations of NRAS on additional cancer hallmarks as well as more mechanistic studies on downstream effector engagement, can shed light on differential functional consequences of these molecular lesions. Further, in vivo animal studies and clinical correlations are warranted to validate them as *bona fide* diagnostic and prognostic biomarkers for CRC. Although NRAS E132K was identified from young-onset CRC cases, large-scale studies and analyses of young-onset versus late-onset CRC datasets are necessary to qualify it as a biomarker for the young-onset subtype.

## Supplementary information

Supplementary Figure 1

## Data Availability

Data generated in this study are available from the corresponding author on reasonable request.
