# Supplementary material for: NRAS mutant E132K identified in young-onset sporadic colorectal cancer and the canonical mutants G12D and Q61K affect distinct oncogenic phenotypes
Source: Sci Rep. 2020 Jul 3;10:11028. doi: 10.1038/s41598-020-67796-8 (PMC7334206; doi:10.1038/s41598-020-67796-8)

NRAS mutant E132K identified in young-onset sporadic colorectal cancer and the canonical mutants G12D and Q61K affect distinct oncogenic phenotypes

Ryan Timothy D. Yu and Reynaldo L. Garcia*

Disease Molecular Biology and Epigenetics Laboratory, National Institute of Molecular Biology and Biotechnology, National Science Complex, University of the Philippines Diliman, Quezon City, 1101, Philippines

**Corresponding Author:*

Reynaldo L. Garcia, PhD MPhil (*cantab*)

Address: National Institute of Molecular Biology and Biotechnology, Ma. Regidor St., National Science Complex, University of the Philippines Diliman, Quezon City 1101, Philippines
Email: reygarcia@mbb.upd.edu.ph
ORCID iD: 0000-0003-1350-1261

Supplementary Fig. 1. Uncropped Western blot analysis of E-cadherin expression in HCT116 cells overexpressing NRAS mutants. The uncropped blots of E-cadherin (a) and GAPDH (b) are shown with the molecular weight marker. From left to right: Wild Type, Q61K, G12D, E132K, empty vector control.

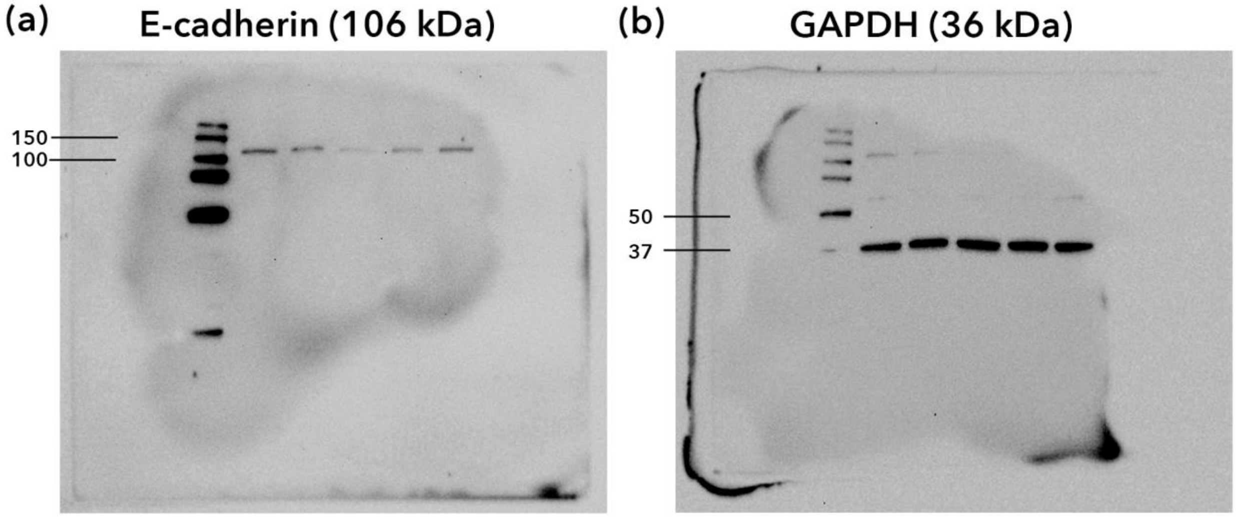

Supplement: Supplementary file 1 — Supplementary Figure 1 [file 41598_2020_67796_MOESM1_ESM.pdf]
